# Techno-functional characterization of gelatin extracted from the smooth-hound shark skins: Impact of pretreatments and drying methods

**DOI:** 10.1016/j.heliyon.2023.e19620

**Published:** 2023-08-29

**Authors:** Ali Salem, Ola Abdelhedi, Haifa Sebii, Fadia Ben Taheur, Nahed Fakhfakh, Mourad Jridi, Nacim Zouari, Frederic Debeaufort

**Affiliations:** a Laboratory of Functional Physiology and Valorization of Bio-resources (LR17ES27), Higher Institute of Biotechnology of Beja (ISBB), University of Jendouba, 9000, Beja, Tunisia; b Food Valuation and Safety Analysis Laboratory, Engineering National School of Sfax (ENIS), University of Sfax, Sfax, Tunisia; c High Institute of Applied Biology of Medenine, University of Gabes, Medenine, Tunisia; d Univ. Bourgogne Franche-Comté/Agrosup Dijon, UMR PAM A02.102, Physical-Chemistry of Food and Wine Lab, 1 Esplanade Erasme, 21000, Dijon, France; e IUT Dijon-Auxerre, BioEngineering Department, University of Burgundy, 7 Blvd Docteur Petit Jean, 21078, Dijon Cedex, France

**Keywords:** Gelatin, Smooth-hound shark (*Mustelus mustelus*) skin, Drying, Gel strength, Foaming property, Emulsifying property

## Abstract

Gelatin derived from marine by-products could be an interesting alternative to classic mammalian gelatin. The pretreatment and extraction conditions could influence the size of the resulting peptide chains and therefore their techno-functional properties. Thus, it is important to optimize the production process to get a gelatin for the appropriate applications. Skin pretreatment was done by microwaves or oven-drying and the extracted gelatin was dried by spray- or freeze-drying. Freeze-dried gelatin extracted from untreated skin (FGUS) had the highest gelatin yield (10.40%). Gelatin proximate composition showed that proteins were the major component (87.12–89.95%), while lipids showed the lowest contents (0.65–2.26%). Glycine showed the highest level (299–316/1000 residues) in the extracted gelatins. Proline and hydroxyproline residues of gelatins from untreated skin were significantly higher than those from pretreated skin-gelatin. FTIR spectra were characterized by peaks of the amide A (3430-3284 cm^−1^), B (3000-2931 cm^−1^), I (1636–1672 cm^−1^), II (1539–1586 cm^−1^) and III (1000–1107 cm^−1^). Spray-drying decreased the gelling properties of gelatins, since it reduced gelling and melting temperatures compared to freeze-drying. Skin pretreatment significantly reduced the gel strength of gelatin by about 50–100 g depending on the gelatin drying method. The FGUS showed better surface properties compared to other gelatins. The highest emulsion activity index (39.42 ± 1.02 m^2^/g) and foaming expansion (172.33 ± 2.35%) were measured at 3% FGUS. Therefore, the promising properties of freeze-dried gelatin derived from untreated skin, gave it the opportunity to be successfully used as a techno-functional ingredient in many formulations.

## 1 Introduction

Gelatin is a well-known biopolymer obtained by denaturing and partially hydrolyzing collagen from connective tissues and bones of animals [1]. It is characterized by its rheological and emulsifying properties. The emulsifying property provided foaming potential, while the viscoelastic properties allowed water retention, cohesion, viscosity and gelling properties. The main important quality of gelatin is its ability to form transparent and thermo-reversible gels, as well as edible coatings. Thus, gelatins are employed in various industries, primarily those of food, pharmaceuticals and cosmetics [2]. The most common source of gelatin was the bones and skin of pigs and cattle [3]. Many studies reported that gelatin comes from pork skin (80%), cattle skin (15%), and pork and cattle bones (5%) [4]. Nevertheless, the use of mammalian gelatins was restricted by many populations due to religious or cultural considerations and earlier worries about food safety following the emergence of zoonotic illnesses in cattle, which prompted a quest for alternative gelatin sources [5]. Fish bones, skins and scales are examples of marine byproducts that are available in large quantities and can be a significant alternative source for the manufacturing of gelatin [6,7]. Tiger tooth croaker [8], Amur sturgeon [9] and Spanish mackerel [10] were among the fish species studied for gelatin extraction using acid pretreatment and heating processes.

One of the most important elements in the proper use of marine by-products was their quality, since they were subjected to rapid microbial spoilage and enzymatic degradation even at low temperature [11]. Therefore, it was crucial to study the techniques for their stabilization and their impact on the quality of the extracted gelatin. In fact, the degree of collagen triple helix cleavage and molecular mass distribution, which were impacted by the processing conditions, determine the techno-functional properties of gelatin [12,13].

Fish skins were mainly stabilized by freezing. However, many studies reported the extraction of gelatin from dried fish skins. Amiza et al. [13] investigated the effects of drying and freezing the skin on the yield, chemical composition, color, gel strength and amino acids composition of the gelatin extracted from cobia (*Rachycentron canadum*) skin. Pranoto et al. [14] characterized the gelatin extracted from sun-dried or fresh skin of seawater fish. It was reported that dried fish skin was better for gelatin production compared to the frozen fresh skin. Giménez et al. [15] reported that the qualities of the raw collagenous material may change during the freezing procedure. Moreover, Amiza et al. [13] showed that drying fish skin enhanced the gel strength of the gelatin relative to gelatin derived from frozen fresh skin.

After gelatin extraction, the main methods used for drying were freeze- and spray-drying, which could affect differently the gelatin's solubility [16]. The characteristics of freeze-dried gelatins included low bulk density, good porosity and flavor. However, the process took relatively a long time and required a lot of energy. On the other hand, compared to freeze-drying, spray-drying enhanced the functional characteristics of gelatin [16,17].

The smooth-hound shark (*Mustelus*, Triakidae), a cartilaginous fish, was widely distributed in the Mediterranean Sea and continental shelves of the eastern Atlantic Ocean [18]. This cartilaginous biomass is readily available in Tunisia from winter to summer, and during processing a significant quantity of by-products was generated that could be valued. Ahmad et al. [19] reported that several shark-derived active substances could have many commercial applications. In the same context, shark cartilage was reported to be used as a source of marine collagen, such us collagen type II and several types of proteoglycans. Also, it was mentioned that shark derived proteins with a molecular mass of 14 kDa were shown to be benefit for several immune diseases such as dendritic cell-mediated T-cell stimulation and induction of desirable immune responses [20]. Besides, according to Ahmed et al. [19] the liver of *Squalus acanthias* (dogfish shark) was used to extract proteins with a high ability to stop angiogenesis and to inhibit the tumor growth.

The current study aimed to extract gelatin with the optimum technological and functional qualities with respect to the appropriate pretreatment and drying technique. Thus, two pretreatments of the fish skin were used: (i) microwave-drying (350 W; 5 min) and (ii) oven-drying (105 °C; 24 h). The extracted gelatins were then dried using spray- or freeze-drying techniques and their physico-chemical and techno-functional characteristics were studied.

## 2 Materials and methods

### 2.1 Experimental reagents

NaOH, acetic acid, phosphate buffer (with 0.1 M K_2_HPO_4_ and KH_2_PO_4_), sodium dodecyl sulfate and NaCl were purchased from Sigma-Aldrich (St. Louis, USA).

### 2.2 Gelatin extraction from smooth-hound skin

The smooth-hound (*M. mustelus*) skin was obtained from a fish market (Sfax, Tunisia) in February 2020. When it arrived, the viscera and blood were eliminated, and the skin tissue was manually scraped with a sharp knife. The gelatin was extracted after skin pretreatment. In fact, the skin was pretreated using: (i) microwave (MW25S; LG Electronics Tianjin Appliance Co., Ltd., China) at 350 W for 5 min or (ii) oven (Karl Kolb D-6072, Dreieich, Germany) at 105 °C during 24 h.

The gelatin was extracted from the fish skin using the method previously described by Jridi et al. [21]. The method was adapted to extract gelatin from the fish skin. Fish skin was placed in a 0.05 M NaOH solution (1/5: m/v) with stirring for 4 h, and the solution was changed every 1 h. Then, the pH was adjusted to 3.0 with acetic acid and the mixture was stirred gently during 18 h. The pH was neutralized and the collagen solution was stirred at 50 °C for 24 h using a water bath at 50 °C. After centrifuging at 6000×*g* for 20 min at 25 °C, the supernatant was dried using spray- or freeze-drying techniques. Six gelatins were produced based on the skin pretreatment and gelatin drying techniques. FGUS, FGMS and FGOS were the freeze-dried gelatins from untreated, microwaves-pretreated and oven-pretreated skin, respectively. While, SGUS, SGMS and SGOS were the spray-dried gelatins from untreated, microwaves-pretreated and oven-pretreated skin, respectively.

The gelatin extraction yield was calculated using equation (1).

(1) $$Yield\;\left(\%\right)=\left(\frac{dried\;gelatin\;\left(g\right)}{fresh\;fish\;skin\;\left(g\right)}\right)\times 100$$

### 2.3 Proximate analyses

The proteins (method 981.10), moisture (method 142.0), lipids (method 948.22) and ash (method 942.05) of different gelatins from smooth hound skins were measured using the AOAC methods [22].

### 2.4 Molecular mass (MM) distribution

Using a fast protein liquid chromatography (GE Healthcare Europe GmbH, Munich, Germany) equipped using a silica gel packed in a TSKgel G2000SWXL column (7.8 mm id × 30 cm), the MM analysis of gelatin was done as previously reported [23]. Elution was performed using 0.1 M phosphate buffer; 0.2 M sodium chloride. The standard markers were blue dextran (2000 kDa), apoferritin (443 kDa), β-amylase (200 kDa) and bovine serum albumin (66 kDa) (Sigma-Aldrich). The gelatins and standard markers were loaded into the column at 5 mg/ml. The MM was calculated from the standard curve.

### 2.5 Amino acid composition

Each sample of gelatin (5 mg/ml) was hydrolyzed using 6 N HCl at 110 °C for 24 h before being filtered through a membrane filter (0.45 m). The treated sample (10 μl) was then derivatized using 6-aminoquinolyl-N-hydroxysuccinimidyl carbamate (Sigma-Aldrich). The amino acid composition was determined using a Waters 2996 Separation Module (Photodiode Array Detector) equipped with a Waters AccQTag amino acids analyzing column (Nova-Pak C18, 150 × 3.9 mm). Based on the peak area and compared to those of standard amino acids (Accq tag chemistry kits -WAT088122), the amino acid composition was estimated and expressed as number of amino acid residues/100 amino acids in each sample.

### 2.6 Color measurement

The L*, a* and b* of each gelatin powder were measured using a colorimeter (Konica Minolta, Osaka, Japan). Total color difference (ΔE) and color saturation (C*) were calculated using equations (2), (3).

(2) $$\Delta E=\sqrt{{{(L}^{*}-L_{c}^{*})}^{2}+{\left(a^{*}-a_{c}^{*}\right)}^{2}+{{(b}^{*}-b_{c}^{*})}^{2}}$$

(3) $$C^{*}=\sqrt{{a^{*}}^{2}+{b^{*}}^{2}}$$

where L*, a* and b* are relative to the gelatins extracted from the pretreated skin; L*_c_, a*_c_ and b*_c_ are relative to gelatins extracted from the untreated skin.

### 2.7 Fourier transform infrared (FTIR) analysis

A FTIR spectrometer (Nicolet, Ettlingen, Germany) equipped with an attenuated total reflection (ATR) accessory was used to conduct infrared analysis on each gelatin sample [24]. The infrared beam incident at a 45° angle on the internal reflection crystal, which was composed of pure diamond zinc selenide. At normal temperature, the spectra's measurement range was 4000–500 cm^−1^. At a resolution of 4 cm^−1^, automatic signals were gathered in 32 scans at 25 °C. The data gathering software package OPUS 3.0 was used to analyze spectral data (Bruker, Ettlingen, Germany).

### 2.8 Gelling and melting points

As previously described by Salem et al. [16], the gelling point was measured. A ratio of 6.67% (m/v) of distilled water was used to dissolve the gelatin powder. Ten ml of the mixture were placed in pots at 4 °C up to being a gel. Using a numeric thermometer (Termoprodukt, Bielawa, Poland), the gelation temperature was visually monitored every 15 min until it reached the temperature at which the solution gelled and that was noted as the gelling point. The melting temperature was then calculated by heating the same solution until the first drop appeared.

### 2.9 Textural profile analysis (TPA)

The TPA was determined using a texture analyzer (Lloyd Instruments, West Sussex, UK) as reported by Elavarasan et al. [8] with some modifications. The gelatin was dissolved in distilled water (6.67% (m/v)) for 30 min at 60 °C and then kept at 4 °C for 18 h. The analyzed gel had a height of 2.7 cm and a diameter of 3.8 cm. Using a 12-mm cylindrical probe with a speed of 5 mm/s and trigger force of 0.05 N, the gel was compressed twice to 30% of its initial height.

### 2.10 Water holding capacity (WHC) and oil holding capacity (OHC)

With a few minor adjustments, the Tkaczewska et al. [25] method was used to measure the WHC and OHC. In 3 ml of distilled water or sunflower oil, 0.2 g gelatin was dissolved. The solution was blended for 10 min with a Vortex mixer, centrifuged at 10,000×*g* for 15 min, and the supernatant was then drained. The WHC, which is given as ml of water bound per g of gelatin, was calculated by measuring the difference between the initial volume of the added water and the volume of the recovered supernatant. The OHC was calculated in the same way and expressed as ml of oil bound per g of gelatin.

### 2.11 Emulsifying property

The method of Tkaczewska et al. [25] with slight modifications was used to determine the emulsifying property. At final concentrations of 1, 2 and 3% (m/v), the gelatin was dissolved in distilled water at 60 °C for 30 min. An Ultra-Turax homogenizer (IKA, Staufen, Germany) was used to homogenize 30 ml of gelatin solution with 10 ml of maize oil. At 0 and 10 min after homogenization, aliquots of the emulsion (50 μl) were removed from the bottom of the container and diluted 100 times with 0.1% sodium dodecyl sulfate solution. A vortex mixer was used to completely homogenize the solutions for 10 s. An UV-VIS spectrophotometer (PG Instruments Ltd, Wibtoft, UK) was used to measure the diluted solutions’ absorbance at 500 nm. As soon as possible (A0), the absorbance is measured. Equations (4) and (5) were used to determine the emulsifying activity index (EAI) and the emulsion stability index (ESI) from the absorbance measurements taken immediately (A_0_) and 10 min (A_10_) after emulsion formation.

(4) $$EAI\;\left(m^{2}/g\right)=\left(\frac{2\times 2.303\times A0\times N}{\varphi \times C\times 10,000}\right)$$

where N is the dilution factor; C is the protein concentration (g/ml) and φ is the oil volumetric fraction (0.25).

(5) $$ESI\;\left(\min\right)=\left(\frac{A0\times 10}{A0-A10}\right)$$

### 2.12 Foaming property

The Tkaczewska et al. [25] method with some modifications was used to measure the foaming property. The gelatins were dissolved in distilled water at 60 °C for 30 min to produce the gelatin solutions at various concentrations of 1, 2 and 3% (m/v). The foam was produced by homogenizing 30 ml of each gelatin solution for 1 min in a Moulinex R62 homogenizer. A graduated cylinder was used to measure the overall volume. equation (6) was used to calculate the foaming capacity, which was the foam expansion immediately following 1 min of homogenization.

(6) $$Foaming\;expansion\;\left(\%\right)=\left(\frac{V1-V0}{V0}\right)\times 100$$

where V_0_ is the initial volume before homogenization (30 ml); V_1_ is the total volume after 1 min of homogenization.

The whipped sample was additionally permitted to stand at room temperature for 15, 30, and 60 min, and the residual volume that represented the foam stability was estimated using equation (7).

(7) $$Foam\;stability\;\left(\%\right)=\left(\frac{V2-V0}{V1-V0}\right)\times 100$$

where V_0_ is the initial volume before homogenization (30 ml); V_1_ is the total volume after 1 min of homogenization; V_2_ is the residual volume at different rest times of the foam.

### 2.13 Statistical analysis

ANOVA analysis was done with SPSS version 18.0, professional edition. Differences were considered significant at *p* < 0.05. For three gelatin samples (*n* = 3), each measurement was made at least three times.

## 3 Results

### 3.1 Chemical characterization and amino acid composition

The gelatin yield is an important parameter, which should be determined especially when the gelatin will be produced on an industrial scale [26]. Table 1 shows the impact of skin pretreatment and drying techniques on the gelatin yield. The gelatin yield varied from 7.83 to 10.40% depending on the different treatments. The gelatin yields from pretreated skin were lower than those from the untreated skin.

**Table 1:** Effect of drying methods on the chemical composition of gelatins extracted from smooth-hound shark skin.

Skin pretreatment method	Gelatin drying method	Gelatins*	**Yield (g/100 g)**	**Fat (g/100 g)**	**Protein (g/100 g)**	**Ash (g/100 g)**	Moisture (g/100 g)
**Untreated**	**Freeze-drying**	**FGUS**	10.40 **±** 0.50^aA^	1.52 ± 0.48^aA^	88.56 ± 3.12^aA^	3.36 ± 0.24^bA^	6.56 ± 0.72^aA^
**Microwaves**		**FGMS**	7.85 ± 0.40^bA^	0.65 ± 0.05^aA^	87.98 ± 4.10^aA^	5.69 ± 0.36^aA^	5.68 ± 0.12^aA^
**Oven**		**FGOS**	8.67 ± 0.61^bA^	0.97 ± 0.01^aA^	88.46 ± 2.25^aA^	5.90 ± 0.25^aA^	4.67 ± 0.15^bA^
**Untreated**	**Spray-drying**	**SGUS**	8.76 ± 0.98^aB^	2.26 ± 0.25^aA^	89.95 ± 2.25^aA^	2.22 ± 0.46^bB^	5.57 ± 0.23^aA^
**Microwaves**		**SGMS**	7.83 ± 1.12^aA^	0.89 ± 0.12^aA^	87.12 ± 1.28^aA^	5.62 ± 0.56^aA^	5.78 ± 0.24^aA^
**Oven**		**SGOS**	7.98 ± 0.87^aB^	0.78 ± 0.07^aA^	88.26 ± 1.85^aA^	4.89 ± 0.27^aA^	4.26 ± 0.86^aA^

*FGUS: Freeze-dried Gelatin from the Untreated Skin; FGMS: Freeze-dried Gelatin from the Microwaves-pretreated Skin; FGOS: Freeze-dried Gelatin from the Oven-pretreated Skin; SGUS: Spray-dried Gelatin from the Untreated Skin; SGMS: Spray-dried Gelatin from the Microwaves-pretreated Skin; SGOS: Spray-dried Gelatin from the Oven-pretreated Skin. ^a,b^ Different lower case letters in the same column within the same drying method indicate significant differences (*p* < 0.05). ^A,B^ Different capital letters in the same column within the same pretreatment method indicate significant differences (*p* < 0.05).

Chemical composition of extracted gelatins was also presented in Table 1. The gelatins were characterized by a relatively low contents of ash (2.22–5.90%), moisture (4.26–6.56%) and fat (0.65–2.26%). Gelatins obtained from untreated skin revealed higher fat content (1.52–2.26%) than gelatins extracted from dried skins. On the other hand, proteins represented the major component that varied between 87.12 and 89.95%.

The amino acid content (residues/1000 residues) of the various types of gelatin was presented in Table 2. The imino acids in SGUS, SGMS and SGOS were about 135, 130 and 128 residues/1000 residues, respectively. Likewise, in FGUS, FGMS and FGOS, they were about 138, 133 and 130 residues/1000 residues, respectively.

**Table 2:** Amino acid composition (number of residues/1000 residues) of gelatins extracted from smooth-hound shark skin.

Amino acid	Gelatins*					
	FGUS	FGMS	FGOS	SGUS	SGMS	SGOS
Hyp	77.55	74.36	73.57	76.15	71.36	71.28
Asx	38.25	40.35	40.25	36.95	34.56	41.02
Ser	38.75	39.72	36.85	38.45	40.48	38.43
Glx	56.45	58.38	57.81	55.95	52.32	51.75
Gly	315.35	299.36	300.04	316.86	308.74	301.28
His	17.65	17.62	18.68	18.45	20.35	18.50
Arg	31.15	34.36	32.68	32.45	35.36	32.29
Thr	38.35	41.25	40.09	37.65	39.12	35.08
Ala	64.15	66.44	67.21	64.05	60.78	64.44
Pro	60.15	58.35	57.02	59.05	58.69	57.28
Cys	1.15	2.01	3.04	0.95	1.85	2.28
Tyr	8.65	9.95	9.79	7.85	10.80	9.20
Val	83.65	84.68	82.69	83.35	83.36	87.60
Met	8.55	9.15	8.88	7.75	8.25	9.00
Lys	16.35	17.23	18.65	18.55	23.25	22.48
Leu	37.35	38.23	37.87	37.05	38.88	38.04
Ileu	71.75	73.20	75.73	74.55	75.26	79.19
Phe	34.75	35.36	39.15	35.88	36.60	39.86
Total amino acids	1000	1000	1000	1000	1000	1000

*FGUS: Freeze-dried Gelatin from the Untreated Skin; FGMS: Freeze-dried Gelatin from the Microwaves-pretreated Skin; FGOS: Freeze-dried Gelatin from the Oven-pretreated Skin; SGUS: Spray-dried Gelatin from the Untreated Skin; SGMS: Spray-dried Gelatin from the Microwaves-pretreated Skin; SGOS: Spray-dried Gelatin from the Oven-pretreated Skin. The aspartic and glutamic acids include, respectively, asparagine and glutamine, Asx = Asp + Asn; Glx = Glu + Gln.

### 3.2 Distribution of molecular mass (MM)

The gelatins’ MM distribution was shown in Table 3. There was no discernible difference between the two skin pre-treatments in terms of the MM distribution of gelatins. The major fraction representing 67.47% for FGUS and 65.82% for SGUS ranged from 10 to 120 kDa. The second fraction has a MM between 120 and 200 kDa representing 27.58–29.38% for the gelatins derived from the untreated skin. However, skin pretreatments decreased these fractions level, while the level of peptides with low MM (<10 kDa) increased significantly from less than 1% to more than 32% (Table 3).

**Table 3:** Molecular mass (MM) distribution (%) of gelatins extracted from smooth-hound shark skin.

MM	Gelatins*					
	FGUS	FGMS	FGOS	SGUS	SGMS	SGOS
>200 kDa	4.00	1.25	1.62	4.50	2.32	2.02
120–200 kDa	27.58	12.50	14.20	29.38	15.30	12.80
10–120 kDa	67.47	50.05	50.48	65.82	46.68	52.68
<10 kDa	0.95	36.20	33.70	0.30	35.70	32.50

*FGUS: Freeze-dried Gelatin from the Untreated Skin; FGMS: Freeze-dried Gelatin from the Microwaves-pretreated Skin; FGOS: Freeze-dried Gelatin from the Oven-pretreated Skin; SGUS: Spray-dried Gelatin from the Untreated Skin; SGMS: Spray-dried Gelatin from the Microwaves-pretreated Skin; SGOS: Spray-dried Gelatin from the Oven-pretreated Skin.

### 3.3 Analysis using the fourier transform infrared (FTIR)

**Fig. 1:**
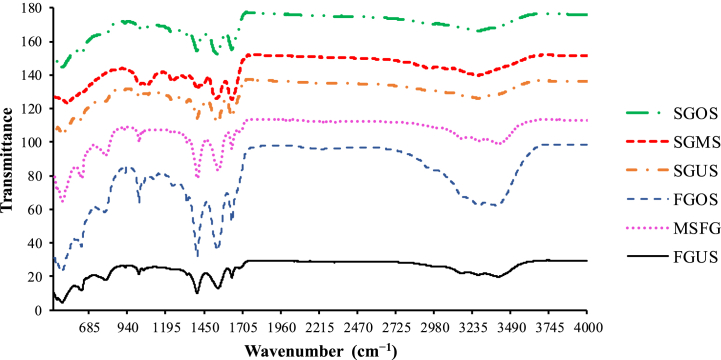
FTIR spectra of gelatins extracted from smooth-hound shark skin.

Table 4 and Fig. 1 showed the FTIR spectrum data for the studied gelatins. As reported for previous fish gelatins, the amide A, B, I, II and III peaks distinguished the FTIR spectra. The peaks of amide A (3284–3430 cm^−1^) were related to the stretch of the NH group, while the peaks of amide B (293–3000 cm^−1^) were associated to the interaction of –NH_2_ or –CH_2_ groups between peptide chains. Amides I (1636–1672 cm^−1^) and II (1539–1586 cm^−1^) were associated to vibrations of C═O stretch and N–H flexion coupled with C–N stretch, respectively. The absorption of the amide III (1000–1107 cm^−1^) resulted from the combination of N–H flexion and C–N stretch.

**Table 4:** FTIR spectra of gelatins extracted from smooth-hound shark skin.

Gelatins*	Wavenumbers (cm^−1^)				
	Amide A	Amide B	Amide I	Amide II	Amide III
**FGUS**	3417	3000	1637	1549	1000
**FGMS**	3430	2983	1639	1550	1021
**FGOS**	3427	2974	1640	1549	1022
**SGUS**	3284	2966	1636	1540	1002
**SGMS**	3305	2957	1672	1586	1107
**SGOS**	3297	2931	1635	1539	1081

*FGUS: Freeze-dried Gelatin from the Untreated Skin; FGMS: Freeze-dried Gelatin from the Microwaves-pretreated Skin; FGOS: Freeze-dried Gelatin from the Oven-pretreated Skin; SGUS: Spray-dried Gelatin from the Untreated Skin; SGMS: Spray-dried Gelatin from the Microwaves-pretreated Skin; SGOS: Spray-dried Gelatin from the Oven-pretreated Skin.

Table 4 shows a small shift in the wavenumbers of the amide bonds of the different extracted gelatins. Amide-A bonds of spray-dried gelatins presented the lowest wavenumbers (3284–3305 cm^−1^) compared to those (3417–3430 cm^−1^) of freeze-dried gelatins.

### 3.4 Color analysis

The L*, a*, b*, ΔE, C* of the gelatins were presented in Table 5. The freeze-dried gelatins displayed the lowest L* values, when compared to the spray-dried gelatins. The FGUS presented the lowest L* (88.72), while, the SGUS represented the clearest gelatin (L* = 94.63). All of the gelatins’ a* values were negative and not far from zero, indicating a little change in hue toward green. In addition, the b* values of the freeze-dried gelatins were lower than those of the spray-dried gelatins, indicating a decrease in their yellowness. Table 5 also shows the chroma (C*) and total color difference (ΔE) of the different gelatins. Spray-dried gelatins showed the highest ΔE values (2.04 and 4.15 for SGMS and SGOS, respectively). In addition, spray-drying increased the saturation in color of extracted gelatins.

**Table 5:** Color properties of gelatins extracted smooth-hound shark skin.

Gelatins*	L*	a*	b*	C*	ΔE
**FGUS**	88.72 ± 2.13^aB^	−0.26 ± 0.01^aA^	5.99 ± 0.24^aA^	5.99 ± 0.04^aA^	–
**FGMS**	90.86 ± 0.56^aB^	−0.66 ± 0.02^cB^	4.28 ± 0.48^bA^	4.33 ± 0.24^bA^	2.76 ± 0.46^aA^
**FGOS**	89.89 ± 0.98^aB^	−0.41 ± 0.02^bA^	4.91 ± 0.59^aB^	4.92 ± 0.04^bB^	1.17 ± 0.48^bB^
**SGUS**	94.63 ± 1.20^aA^	−0.27 ± 0.01^aA^	6.34 ± 0.42^cA^	3.35 ± 0.28^cB^	–
**SGMS**	93.58 ± 2.35^aA^	−0.23 ± 0.02^aA^	5.10 ± 0.36^bA^	5.10 ± 0.12^bA^	2.04 ± 0.76^aA^
**SGOS**	93.39 ± 2.47^aA^	−0.36 ± 0.04^bA^	7.30 ± 0.58^aA^	7.30 ± 0.19^aA^	4.15 ± 0.89^bA^

*FGUS: Freeze-dried Gelatin from the Untreated Skin; FGMS: Freeze-dried Gelatin from the Microwaves-pretreated Skin; FGOS: Freeze-dried Gelatin from the Oven-pretreated Skin; SGUS: Spray-dried Gelatin from the Untreated Skin; SGMS: Spray-dried Gelatin from the Microwaves-pretreated Skin; SGOS: Spray-dried Gelatin from the Oven-pretreated Skin. ^a,b,c^ Different lower case letters in the same column within the same drying method indicate significant differences (*p* < 0.05). ^A,B^ Different capital letters in the same column within the same pretreatment method indicate significant differences (*p* < 0.05).

### 3.5 Melting and gelling temperatures

Table 6 showed the gelling and melting temperatures. Gelling temperature of the extracted gelatins ranged from 11.05 to 14.95 °C. Untreated skins’ gelatins had a greater gelling temperature than those from pretreated skins. The highest gelling temperature was obtained for FGUS, which became gel at 14.95 °C. Similarly, skin-pretreatment affected melting temperature, which significantly decreased (*p* < 0.05) compared to the gelatin from untreated-skins. Likewise, the drying method of gelatin influenced the melting and gelling temperatures. Gelling and melting temperature of freeze-dried gelatins were higher than those of spray-dried gelatins, which suggest that spray-drying decreased the gelling properties of gelatins.

**Table 6:** Thermal and texture properties of gelatins extracted from smooth-hound shark skin.

Gelatins*	Gelling point (°C)	Melting point (°C)	Gel strength (g)	Cohesiveness	Springiness (mm)	Chewiness (N × mm)
**FGUS**	14.95 ± 0.42^aA^	18.56 ± 0.85^aA^	198.25 ± 2.35^aA^	0.21 ± 0.01^bA^	14.46 ± 0.87^aA^	30.97 ± 1.24^aA^
**FGMS**	13.56 ± 0.11^aA^	18.48 ± 0.12^aA^	150.26 ± 3.26^bA^	0.32 ± 0.02^aA^	13.52 ± 0.56^aA^	9.24 ± 1.05^bA^
**FGOS**	12.80 ± 0.15^bA^	17.90 ± 1.77^aA^	98.56 ± 1.25^cA^	0.20 ± 0.01^bA^	12.56 ± 0.78^bA^	5.23 ± 0.58^cB^
**SGUS**	14.80 ± 0.48^aA^	19.54 ± 0.47^aA^	196.45 ± 2.48^aA^	0.21 ± 0.01^bA^	13.15 ± 0.74^aA^	28.12 ± 1.47^aA^
**SGMS**	11.05 ± 0.70^bB^	18.25 ± 0.49^aA^	130.23 ± 1.27^bB^	0.28 ± 0.04^aA^	13.00 ± 1.01^aA^	9.30 ± 0.69^bA^
**SGOS**	11.65 ± 0.40^aA^	18.88 ± 0.56^aA^	93.56 ± 3.26^cA^	0.23 ± 0.07^bA^	11.75 ± 0.93^bA^	9.39 ± 0.89^bA^

*FGUS: Freeze-dried Gelatin from the Untreated Skin; FGMS: Freeze-dried Gelatin from the Microwaves-pretreated Skin; FGOS: Freeze-dried Gelatin from the Oven-pretreated Skin; SGUS: Spray-dried Gelatin from the Untreated Skin; SGMS: Spray-dried Gelatin from the Microwaves-pretreated Skin; SGOS: Spray-dried Gelatin from the Oven-pretreated Skin. ^a,b,c^ Different lower case letters in the same column within the same drying method indicate significant differences (*p* < 0.05). ^A,B^ Different capital letters in the same column within the same pretreatment method indicate significant differences (*p* < 0.05).

### 3.6 Technological characteristics

#### 3.6.1 Texture analysis

Table 6 shows the textural characteristics of the gelatin gels in terms of gel strength, cohesiveness, springiness and chewiness. The skin pretreatments significantly decreased (*p* < 0.05) the gel strength and the chewiness of gelatin gels, which was consistent with gelling and melting points. In addition, the skin oven-drying pretreatment significantly decreased the gel strength (*p* < 0.05) compared to the microwaves-drying pretreatment. At the same context, the SGMS's gel strength value was reduced by 13% when compared to the FGMS.

#### 3.6.2 Foaming properties

Table 7 shows the foaming potential in terms of foam expansion (FE) and foam stability (FS) of the extracted gelatins. The FE and FS of all gelatin solutions increased with increasing gelatin concentration. The highest FE was measured for FGUS at 3% concentration (172.33%), while the lowest value was measured for SGOS at 1% concentration (150.26%). Spray-dried gelatins showed the lowest FS (144.59–161.25%) when compared to freeze-dried gelatins.

**Table 7:** Emulsifying and foaming properties of gelatins extracted from smooth-hound shark skin.

Gelatins*	(g/100 ml)	EAI (m^2^/g)	ESI (min)	Foaming expansion (%)	Stability at 30 min (%)	Stability at 60 min (%)
**FGUS**	**1**	28.56 ± 0.26^aA^	45.74 ± 1.25^aA^	166.56 ± 2.32^aA^	165.32 ± 1.23^aA^	162.33 ± 1.22^aA^
	**2**	34.57 ± 0.85^aA^	34.26 ± 1.56^aA^	169.42 ± 1.26^aA^	168.22 ± 2.02^aA^	167.44 ± 2.10^aA^
	**3**	39.42 ± 1.02^aA^	24.28 ± 0.75^aA^	172.33 ± 2.35^aA^	171.21 ± 3.06^aA^	171.12 ± 1.14^aA^
**FGMS**	**1**	20.15 ± 0.78^bA^	46.58 ± 1.59^aA^	162.53 ± 4.59^aA^	160.66 ± 2.33^aA^	157.56 ± 3.12^aA^
	**2**	25.48 ± 0.94^bA^	37.24 ± 1.86^aA^	165.46 ± 2.45^aA^	163.88 ± 1.15^aA^	159.98 ± 2.18^bA^
	**3**	29.74 ± 1.25^bA^	25.48 ± 1.05^aA^	169.23 ± 1.26^aA^	168.48 ± 1.27^aA^	167.55 ± 1.24^aA^
**FGOS**	**1**	19.54 ± 0.82^cA^	47.85 ± 2.96^aA^	159.22 ± 1.89^bA^	155.27 ± 3.25^bA^	150.75 ± 2.14^bA^
	**2**	24.45 ± 0.45^bA^	35.79 ± 1.86^aA^	163.42 ± 5.68^aA^	157.23 ± 2.74^bA^	152.33 ± 1.41^cA^
	**3**	27.46 ± 0.59^bA^	26.56 ± 0.99^aB^	166.45 ± 1.17^bA^	159.66 ± 2.53^bA^	154.22 ± 2.74^bA^
**SGUS**	**1**	25.73 ± 1.18^aA^	45.82 ± 1.52^aA^	159.63 ± 3.15^aA^	157.25 ± 1.42^aB^	156.53 ± 2.58^aB^
	**2**	30.25 ± 1.03^aB^	33.75 ± 1.08^bA^	160.26 ± 2.59^aB^	159.22 ± 1.07^aB^	157.36 ± 3.06^aB^
	**3**	32.42 ± 1.63^aB^	24.38 ± 1.01^bA^	163.77 ± 2.48^aB^	162.34 ± 2.04^aB^	161.25 ± 2.01^aB^
**SGMS**	**1**	21.75 ± 0.68^bA^	48.75 ± 1.55^aA^	155.62 ± 1.48^aB^	152.59 ± 2.45^aB^	148.59 ± 1.10^bB^
	**2**	25.71 ± 0.99^bA^	39.48 ± 2.22^aA^	158.42 ± 3.49^aB^	153.69 ± 3.25^bB^	150.36 ± 1.21^bB^
	**3**	30.12 ± 0.82^aA^	27.46 ± 1.04^aA^	159.63 ± 3.55^aB^	155.96 ± 1.58^bB^	151.52 ± 2.31^bB^
**SGOS**	**1**	20.41 ± 0.57^bA^	47.79 ± 1.99^aA^	150.26 ± 4.77^bB^	147.20 ± 2.17^bB^	144.59 ± 1.71^bB^
	**2**	25.17 ± 0.48^bA^	37.55 ± 0.75^aA^	152.42 ± 1.58^bB^	150.08 ± 1.18^bB^	148.26 ± 2.84^bA^
	**3**	29.15 ± 0.88^aA^	28.66 ± 1.96^aA^	154.23 ± 3.16^bB^	151.26 ± 1.16^bB^	149.06 ± 2.47^bB^

*FGUS: Freeze-dried Gelatin from the Untreated Skin; FGMS: Freeze-dried Gelatin from the Microwaves-pretreated Skin; FGOS: Freeze-dried Gelatin from the Oven-pretreated Skin; SGUS: Spray-dried Gelatin from the Untreated Skin; SGMS: Spray-dried Gelatin from the Microwaves-pretreated Skin; SGOS: Spray-dried Gelatin from the Oven-pretreated Skin. EAI: Emulsion Activity Index; ESI: Emulsion Stability Index. ^a,b,c^ Different lower case letters in the same column within the same drying method and the same gelatin concentration indicate significant differences (*p* < 0.05). ^A,B^ Different capital letters in the same column within the same pretreatment method indicate significant differences (*p* < 0.05).

#### 3.6.3 Emulsifying properties

The emulsion activity index (EAI) and emulsion stability index (ESI) of the isolated gelatins at various concentrations (1–3%) were also assessed (Table 7). The EAI increased with increasing gelatin concentration as the highest EAI value was measured for FGUS at 3% of gelatin concentration (39.42 m^2^/g). However, the increase in EAI was followed by an important decrease in ESI. Table 7 also shows that spray-dried gelatins and extracted from pretreated skin showed lowest EAI compared to other gelatins.

#### 3.6.4 Oil holding capacity (OHC) and water holding capacity (WHC)

**Fig. 2:**
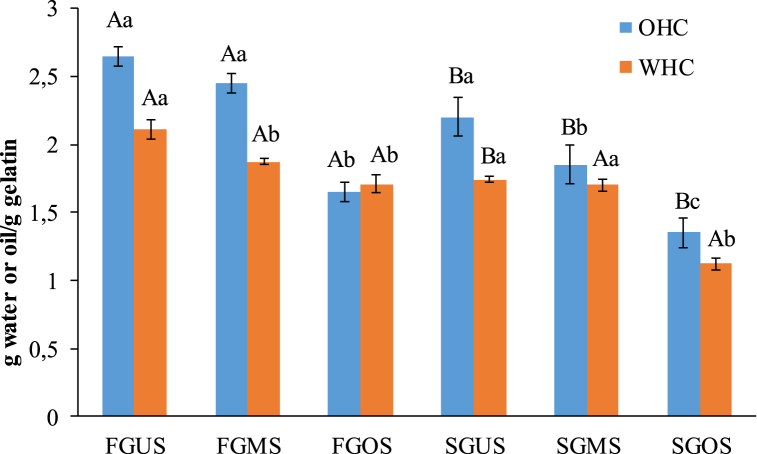
Water holding capacity (WHC) and oil holding capacity (OHC) of gelatins extracted from smooth-hound shark skin. FGUS: Freeze-dried Gelatin from the Untreated Skin; FGMS: Freeze-dried Gelatin from the Microwaves-pretreated Skin; FGOS: Freeze-dried Gelatin from the Oven-pretreated Skin; SGUS: Spray-dried Gelatin from the Untreated Skin; SGMS: Spray-dried Gelatin from the Microwaves-pretreated Skin; SGOS: Spray-dried Gelatin from the Oven-pretreated Skin. ^a,b^ Different lower case letters within the same drying method indicate significant differences (*p* < 0.05). ^A,B^ Different capital letters within the same pretreatment method indicate significant differences (*p* < 0.05).

The OHC and WHC reflected the interactions of gelatin with water and oil, respectively, which influenced the medium texture. Fig. 2 shows that gelatins extracted from pretreated skin had the lowest WHC values compared to gelatins derived from untreated skin. The OHC values of the studied gelatins did not differ significantly (*p* > 0.05), as shown in Fig. 2.

## 4 Discussion

### 4.1 Chemical characterization and amino acid composition

The decrease in gelatin yield could be explained by the negative effect of the high temperature on protein structure. In the same context, Pranoto et al. [14] reported that skin drying led to protein denaturation and promoted protein-protein interactions, which affected collagen swelling and gelatin extraction.

The chemical composition of the different gelatins was determined and the results were presented in Table 1. As mentioned before, the composition revealed that proteins were the highest component for all gelatins. Besides, a little increase in fat content was recorded for the untreated skin gelatin. Fat content was reported to be closely associated with fish species [14]. Likewise, Tkaczewska et al. [25] showed that gelatin extracted by pretreatment with NaOH and ethanol presented high protein content (82.44%). Compared to bovine gelatin [27], the proximate composition of the studied gelatins showed a little increase in ash and fat content, a decrease in moisture content and a similar proteins content.

To determine the effect of the different pretreatments, it is important to study the amino-acid composition of the different gelatins. As reported previously on gelatin extracted from the skin of various fish species [16,21,25,28], the obtained results showed that glycine (299–316) was found to be the main amino acid. The methionine, tyrosine, and cysteine contents of the isolated gelatins were comparatively low, similarly to the results reported by Jridi et al. [21]. Moreover, Table 2 shows that skin pretreatments resulted in a significantly lower (*p* < 0.05) level of glycine and imino acids (proline and hydroxyproline) in gelatin than those measured for the untreated skin. It was reported that collagen telopeptides (cross-linking sites) were rich in imino acids, which played a binding role and participated in the stability of the protein macromolecule [10,29]. The skin's heat exposure during the applied pretreatments might occur to the partial cleavage of these sites and subsequent release of imino acids, as previously reported [30]. It's worthy to note that rheological properties of gelatins were significantly influenced by the imino acids present in gelatin. Low proline and hydroxyproline content in gelatins resulted in a lower melting point and a weaker gel network [28].

### 4.2 Distribution of molecular mass (MM)

The MM distribution of gelatins from *M. mustelus* skin, extracted using various pretreatments and drying methods, was determined and the results were presented in Table 3. Skin pretreatments had an impact on the MM because there was a significant difference in the distribution of the MM between gelatins made from untreated skin and those derived from skin pretreated by microwaves or oven. Likewise, previous studies reported that drying the skin caused collagen degradation, which increased the amount of peptides with low MM [16]. It was reported that textural and functional qualities of gelatin may change when it was partially hydrolyzed to low MM peptides [31]. The obtained results showed that the high fraction was ranged from 10 to 120 kDa, which suggest the presence of the α-chain band as the same for the bovine gelatin [27]. In contrast to the bovine gelatin [27], the highest content of fragments with a MM less than 10 kDa could explain the negative effect of the heat treatment on its functional properties.

### 4.3 Analysis using the fourier transform infrared (FTIR)

The different pretreatment methods, as well as the drying process using for gelatins preparation had certainly an effect on the molecular structure of gelatins. For this reason, the FTIR analysis was realized and the results were presented in Table 4 and Fig. 1. The obtained results were similar to those obtained for bovine gelatin, which indicated that *M. mustelus* gelatin has a similar chemical structure to bovine gelatin [32]. Moreover, these results suggest that spray-drying of gelatin resulted in shorter peptide fragments, which increased the number of NH groups. Compared to untreated skin gelatins, pretreated skin gelatins had amide-III bonds with higher values, which were related to the triple helix structure of gelatin. The obtained results were similar to those of a previous work on the influence of pretreatment and drying methods on the dogfish gelatin properties [16]. The observed shift in the amide bonds wavenumbers might be related to the structural modifications of the collagen following the different heat-treatments [33]. According to previous reports [34,35], drying processes cause collagen to lose its triple helix structure and weaken its peptide chains.

### 4.4 Color analysis

It is major to determine the color of the different gelatins, as that may determine its application after the extraction process [36]. To check the differences in color between the extracted *M. mustelus* gelatins, the color analysis was performed in terms of L*, a*, b*, ΔE, C* (Table 5). Compared to the bovine gelatin, the *M. mustelus* gelatins showed higher values of lightness, redness and yellowness [27]. The obtained results suggested that spray-drying lead to obtain a yellow gelatin powder compared to freeze-dried gelatins. The yellowness color could be related to the high temperature during spray-drying, which could generate the Maillard reactions of amino acids as previously reported [16]. According to Park et al. [37], microwave treatment of duck feet increased the L* and decreased the a* values of the isolated gelatin. Additionally, the obtained results revealed that spray-drying increased the ΔE values as well as the saturation in color. The obtained data were similar to those reported by da Silva Araújo et al. [36], who mentioned that drying conditions affected the color properties and increased the color variation of the gelatin extracted from *Cynoscion acoupa* skin. Likewise, Salem et al. [16] reported comparable results for the dogfish skin gelatin. The chromophores of fish skin may be responsible for the increase in color intensity, suggesting that fish species may impact gelatin color [38].

### 4.5 Melting and gelling temperatures

Melting and gelling temperatures of the studied gelatins were determined and the results were presented in Table 6. The obtained results suggest that high temperature used in spray-drying process lead to decrease the gelling properties of the different gelatins. In contrast, it was reported that freeze-drying of gelatins extracted from shark increased their gelling properties compared to the spray-drying [39]. Hamzeh et al. [35] reported that freeze-drying could participate to the formation of more cross-links in gelatin, which could lead to increase the β and γ-chains fractions. On the other hand, thermal deterioration brought on by the high temperature used in the spray-drying process could explained the decreased gelling ability of the gelatins from pretreated skins [35]. These results were consistent with those found for the distribution of MM and amino-acid composition. Due to their low imino acids content, fish gelatin generally showed lower gelling and melting temperatures than mammalian gelatins [9,33].

### 4.6 Technological characteristics

#### 4.6.1 Texture analysis

The *M. mustelus* gelatin gels (6.67%) were tested for texture analysis (gel strength, cohesiveness, springiness and chewiness) and the results were presented in Table 6. Gel strength gives information about the structure under compression. The elasticity measured the capacity of the gelatin gels structure to support the forces generated by the teeth, as a perception of gels in the mouth, before its broke down by the initial compression. Moreover, chewiness has a relation with the capacity to masticate food before swallowing [40]. The obained results revealed that triple helix state of pretreated skin gelatins was affected by the high temperature that reduced the α- and β-chains fractions, and which gave less rigid gels. From the other hand, it seems that spray-drying of the extracted gelatin decreased its gel strength compared to the freeze-drying method. These results could be related to the high temperature used during drying, which negatively affected the MM distribution, as well as gelling and melting properties of gelatins. In the same context, it was reported that the MM distribution of gelatin affects the gel strength [41]. However, skin pretreatment did not influence the cohesiveness and springiness of the gelatin gels. These findings were comparable to those observed for gelatin extracted from bovine bone using pepsin and acid pretreatment [42]. Gelatin chain interactions, which were a key factor in the creation of the gel network, were impacted by the skin pretreatment [16,43].

#### 4.6.2 Foaming properties

Foaming is a parameter that must be studied since it gave a great information about the food application in food industries. The foaming properties of the studied gelatins (Table 7) remained comparable to other gelatins from other resources [44]. Particularly, the kinetic monitoring of foaming expansion and foaming stability of the studied gelatins was comparable to that obtained from bovine gelatin at concentration of 2 and 3% [45]. The foaming property was dependent on the hydrophobic residues on the protein surface, which increased the FE. The capacity of proteins to quickly adsorb at the air-liquid interface and to create a cohesive and stable film was also linked to their foaming potential [46]. Besides, gelatin with longer chain length and less breakdown likely produced stronger films around the air bubbles. Nevertheless, peptides with low MM were unable to create a well-ordered film at the interface, which led to poor foaming qualities [41,47]. Hence, the various heat processes employed to extract gelatin were very important in determining the gelatin's ability to produce foam.

#### 4.6.3 Emulsifying properties

Emulsifying is one of the functional properties that is much related to the food application of gelatins. The obtained results (Table 7) were in accordance with other results, which reported an increase in EAI accompanied by a decrease in ESI of gelatin solution as the gelatin concentration increased [44]. The measured EAI values were similar to those reported for smooth-hound gelatin (32.39 m^2^/g) [18], cuttlefish (38.43 m^2^/g) [41] and barbel (31.97 m^2^/g) [48]. In addition, emulsifying capacity of gelatin derived from eel skin increased with increasing pH solution [49]. Compared to pigskin gelatin (as a mammalian gelatin) the EAI values of the *M. mustelus* gelatins was the lowest. This could be explained by the high MM peptide chains presented in pigskin gelatin, which lead to increase the stability within the interface protein-oil [50]. The skin pretreatment could affect the ESI of proteins, since heat pretreatment resulted in gelatin denaturation, which altered the solubility and subsequently the emulsifying property of gelatin [30]. The skin pretreatment could affect the emulsifying property of gelatin, because heat pretreatment led to protein denaturation and altered its solubility. Additionally, ESI was closely related to the amino acid composition and molecular mass of gelatins. Indeed, peptides having high MM with high hydrophobic amino acids could improve the EAI and ESI [16]. The results relative to MM distribution showed a decrease in hydrophobic amino-acids for the pretreated skin gelatins, which explained the decrease in their EAI and ESI. In the same context, the increase in peptides of higher MM was reported to contribute to gelatin emulsion stability [21] as was observed for foaming properties.

#### 4.6.4 Oil holding capacity (OHC) and water holding capacity (WHC)

WHC and OHC provide information about the characteristics of protein destined for food applications in food system. These parameters gives an idea about the capacity of gelatins to interact and stabilize the water-oil interfaces. As shown in Fig. 2, the low values obtained for WHC of gelatins derived from pretreated skins could be explained by a decrease in their hydrophilic amino acids and hydroxyproline [49,51]. The OHC values were lower than those reported for gelatins from other sources [44,46]. The OHC resulted from hydrophobic, electrostatic and hydrogen bonds between oil and gelatin. Similarly, Ninan et al. [52] showed that oil retention was closely related to the number of non-polar side chains, as well as the exposure of hydrophobic residues. Spray-dried gelatins showed the lowest WHC and OHC compared to freeze-dried gelatins. These results might be attributed to the high temperature of spray-drying that altered amino acids involved in water and oil retention [35]. Additionally, He et al. [50] illustrated that WHC values of pigskin gelatin was much higher than those obtained for fish skin. In contrast, they found high values of OHC for fish gelatin compared to pigskin gelatin.

## 5 Conclusions

To produce high-quality gelatin, it is important to choose the appropriate pretreatment and drying methods that preserve the functional properties of the gelatin. Thus, six gelatins were extracted from smooth-hound shark skin basing on the pretreatment and drying method. The present study showed that quality of gelatin was affected by the pretreatment of skin (microwaves and oven-drying) and the drying method (spray- and freeze-drying) used. Gelatins derived from untreated skin showed better extraction yield, surface and gelling properties. The skin pretreatment had a detrimental effect on the gelatin quality, which caused gelatin degradation, as shown by FTIR analysis and MM distribution. Freeze-drying was found to be better that spray-drying with respect to the techno-functional characteristics of gelatins. In perspective, potential applications of the extracted gelatin from smooth-hound shark skin could be studied in comparison to conventional gelatin, which can pave the way for the development of new value-added products.

## Author contribution statement

Ali Salem: Conceived and designed the experiments; Performed the experiments; Wrote the paper. Ola Abdelhedi: Contributed reagents, materials, analysis tools or data; Wrote the paper. Haifa Sebii, Fedia Ben Taher: Contributed reagents, materials, analysis tools or data. Nahed Fakhfakh: Analyzed and interpreted the data; Wrote the paper. Mourad Jridi: Contributed reagents, materials, analysis tools or data; Analyzed and interpreted the data; Wrote the paper. Nacim Zouari: Analyzed and interpreted the data; Wrote the paper. Frederic Debeaufort: Contributed reagents, materials, analysis tools or data; Analyzed and interpreted the data.

## Funding statement

No funding was applicable.

## Data availability statement

Data included in article/supplementary material/referenced in article.

## Additional information

No additional information is available for this paper.

## Declaration of competing interest

The authors declare that they have no known competing financial interests or personal relationships that could have appeared to influence the work reported in this paper.

## References

[1] Chuaynukul K., Nagarajan M., Prodpran T., Benjakul S., Songtipya P., Songtipya L. (2018). Comparative characterization of bovine and fish gelatin films fabricated by compression molding and solution casting methods. J. Polym. Environ..

[2] Fallah M., Rouhi M., Soltani M., Mohammadifar M.A., Bahrami R., Davachi S.M., Abbaspourrad A., Mohammadi R. (2021). Physico-mechanical, antimicrobial, and antioxidant properties of gelatin edible films incorporated with olibanum essential oil and sodium hexametaphosphate on the rainbow trout fillet under refrigerated conditions. J. Polym. Environ..

[3] Khiari Z., Rico D., Martin-Diana A.B., Barry-Ryan C. (2017). Valorization of fish by-products: rheological, textural and microstructural properties of mackerel skin gelatins. J. Mater. Cycles Waste Manag..

[4] Lin L., Regenstein J.M., Lv S., Lu J., Jiang S. (2017). An overview of gelatin derived from aquatic animals: properties and modification. Trends Food Sci. Technol..

[5] Zhang Z., Wang Y.M., Qiu Y.T., Chi C.F., Luo H.Y., Wang B. (2022). Gelatin from cartilage of Siberian sturgeon (*Acipenser baerii*): preparation, characterization, and protective function on ultraviolet-A injured human skin fibroblasts. Front. Mar. Sci..

[6] Ali A.M.M., Kishimura H., Benjakul S. (2018). Physicochemical and molecular properties of gelatin from skin of golden carp (*Probarbus Jullieni*) as influenced by acid pretreatment and prior-ultrasonication. Food Hydrocolloids.

[7] Ideia P., Pinto J., Ferreira R., Figueiredo L., Spínola V., Castilho P.C. (2020). Fish processing industry residues: a review of valuable products extraction and characterization methods. Waste Biomass Valorization.

[8] Elavarasan K., Kumar A., Uchoi D., Tejpal C.S., Ninan G., Zynudheen A.A. (2017). Extraction and characterization of gelatin from the head waste of tiger tooth croaker (*Otolithes ruber*). Waste Biomass Valorization.

[9] Alipal J., Pu'Ad N.M., Lee T.C., Nayan N.H.M., Sahari N., Basri H., Mi Idris M.I., Abdullah H.Z. (2021). A review of gelatin: properties, sources, process, applications, and commercialization. Mater. Today: Proc..

[10] Kusumaningrum I., Pranoto Y., Hadiwiyoto S. (2018). Extraction optimization and characterization of gelatine from fish dry skin of Spanish mackerel (*Scomberromorus commersoni*). IOP Conf. Ser. Earth Environ. Sci..

[11] Zhang S.Y., Zhao Y.Q., Wang Y.M., Yang X.R., Chi C.F., Wang B. (2022). Gelatins and antioxidant peptides from Skipjack tuna (*Katsuwonus pelamis*) skins: purification, characterization, and cytoprotection on ultraviolet-A injured human skin fibroblasts. Food Biosci..

[12] Nguyen Le M.L., Le Thi H.N., Nguyen V.T. (2021). Hydrolyzed Karaya gum: gelatin complex coacervates for microencapsulation of soybean oil and curcumin. J. Food Qual..

[13] Amiza M.A., Siti Aishah D. (2011). Effect of drying and freezing of Cobia (*Rachycentron canadum*) skin on its gelatin properties. Int. Food Res. J..

[14] Pranoto Y., Marseno D.W., Rahmawati H. (2011). Characteristics of gelatins extracted from fresh and sun-dried seawater fish skins in Indonesia. Int. Food Res. J..

[15] Giménez B., Gómez-Guillén M.C., Montero P. (2005). Storage of dried fish skins on quality characteristics of extracted gelatin. Food hydrocoll.

[16] Salem A., Fakhfakh N., Jridi M., Abdelhedi O., Nasri M., Debeaufort F., Zouari N. (2020). Microstructure and characteristic properties of dogfish skin gelatin gels prepared by freeze/spray-drying methods. Int. J. Biol. Macromol..

[17] El Amine K.M. (2022). Effect of gelatin drying methods on its amphiphilicity. Foods Raw Mater.

[18] Bougatef A., Balti R., Sila A., Nasri R., Graiaa G., Nasri M. (2012). Recovery and physicochemical properties of smooth hound (*Mustelus mustelus*) skin gelatin. Lebensm. Wiss. Technol..

[19] Ahmed M., Verma A.K., Patel R. (2020). Collagen extraction and recent biological activities of collagen peptides derived from sea-food waste: a review. Sustain Chem Pharm.

[20] Lu W.C., Chiu C.S., Chan Y.J., Mulio A.T., Li P.H. (2023). Characterization and biological properties of marine by-product collagen through ultrasound-assisted extraction. Aquac. Rep..

[21] Jridi M., Nasri R., Salem R.B.S.B., Lassoued I., Barkia A., Nasri M., Souissi N. (2015). Chemical and biophysical properties of gelatins extracted from the skin of octopus (*Octopus vulgaris*). Lebensm. Wiss. Technol..

[22] Aoac (1990).

[23] Jridi M., Nasri R., Lassoued I., Souissi N., Mbarek A., Barkia A., Nasri M. (2013). Chemical and biophysical properties of gelatins extracted from alkali-pretreated skin of cuttlefish (*Sepia officinalis*) using pepsin. Int. Food Res. J..

[24] Abdelhedi O., Nasri R., Jridi M., Kchaou H., Nasreddine B., Karbowiak T., Debeaufort F., Nasri M. (2018). Composite bioactive films based on smooth-hound viscera proteins and gelatin: physicochemical characterization and antioxidant properties. Food Hydrocolloids.

[25] Tkaczewska J., Morawska M., Kulawik P., Zając M. (2018). Characterization of carp (*Cyprinus carpio*) skin gelatin extracted using different pretreatments method. Food Hydrocolloids.

[26] Kim T.K., Ham Y.K., Shin D.M., Kim H.W., Jang H.W., Kim Y.B., Choi Y.S. (2020). Extraction of crude gelatin from duck skin: effects of heating methods on gelatin yield. Poultry Sci..

[27] Aykın-Dinçer E., Koç A., Erbaş M. (2017). Extraction and physicochemical characterization of broiler (*Gallus gallus domesticus*) skin gelatin compared to commercial bovine gelatin. Poultry Sci..

[28] Renuka V., Ravishankar C.N.R., Zynudheen A.A., Bindu J., Joseph T.C. (2019). Characterization of gelatin obtained from unicorn leatherjacket (*Aluterus monoceros*) and reef cod (*Epinephelus diacanthus*) skins. Lebensm. Wiss. Technol..

[29] Jridi M., Nasri M. (2016). Gélatines de Poisson: préparation, propriétés et applications. Nutr. Sant.

[30] Nagarajan M., Benjakul S., Prodpran T., Songtipya P., Kishimura H. (2012). Characteristics and functional properties of gelatin from splendid squid (*Loligo formosana*) skin as affected by extraction temperatures. Food hydrocoll.

[31] Abdelhedi O., Nasri R., Mora L., Toldrá F., Nasri M., Jridi M. (2017). Collagenous proteins from black-barred halfbeak skin as a source of gelatin and bioactive peptides. Food Hydrocolloids.

[32] Firdausiah S., Madya N., Rapak M.T. (2021). Chemical properties of fish gelatin from skin and bone of yellowfin tuna (*Thunnus albacares*). Indonesia Chimica Acta.

[33] Sinthusamran S., Benjakul S., Hemar Y., Kishimura H. (2018). Characteristics and properties of gelatin from seabass (*Lates calcarifer*) swim bladder: impact of extraction temperatures. Waste Biomass Valorization.

[34] Chen C., Liu F., Yu Z., Ma Y., Goff H.D., Zhong F. (2020). Improvement in physicochemical properties of collagen casings by glutaraldehyde cross-linking and drying temperature regulating. Food Chem..

[35] Hamzeh A., Benjakul S., Sae-Leaw T., Sinthusamran S. (2018). Effect of drying methods on gelatin from splendid squid (*Loligo formosana*) skins. Food Biosci..

[36] da Silva Araújo C., Pino-Hernández E., Batista Souza, T J., Sarkis Peixoto, Joele M.R., de Arimateia Rodrigues do Rego J., Lourenço Henriques, L D F. (2021). Optimization of fish gelatin drying processes and characterization of its properties. Sci. Rep..

[37] Park J.H., Choe J.H., Kim H.W., Hwang K.E., Song D.H., Yeo E.J., Kim H.Y., Choi Y.S., Lee S.H., Kim C.J. (2013). Effects of various extraction methods on quality characteristics of duck feet gelatin. Food Sci. Anim. Resour..

[38] George N., Zynudheen A.A., Anu J., Binsi P.K., Joshy C.G. (2015). Effect of pretreatment conditions and bleaching on physico-chemical and functional properties of gelatin prepared from cuttlefish skin. Indian J. Fish..

[39] Sae-Leaw T., Benjakul S., O'Brien N.M. (2016). Effect of pretreatments and drying methods on the properties and fishy odor/flavor of gelatin from seabass (*Lates calcarifer*) skin. Dry. Technol..

[40] Sila A., Martinez-Alvarez O., Krichen F., Gómez-Guillén M.C., Bougatef A. (2017). Gelatin prepared from European eel (*Anguilla anguilla*) skin: physicochemical, textural, viscoelastic and surface properties. Colloids Surf. A Physicochem. Eng. Asp..

[41] Balti R., Jridi M., Sila A., Souissi N., Nedjar-Arroume N., Guillochon D., Nasri M. (2011). Extraction and functional properties of gelatin from the skin of cuttlefish (*Sepia officinalis*) using smooth hound crude acid protease-aided process. Food Hydrocolloids.

[42] Cao S., Wang Y., Xing L., Zhang W., Zhou G. (2020). Structure and physical properties of gelatin from bovine bone collagen influenced by acid pretreatment and pepsin. Food Bioprod. Process..

[43] Ma Y., Zeng X., Ma X., Yang R., Zhao W.A. (2019). Simple and eco-friendly method of gelatin production from bone: one-step biocatalysis. J. Clean. Prod..

[44] Zarai Z., Balti R., Sila A., Ali Y.B., Gargouri Y. (2016). *Helix aspersa* gelatin as an emulsifier and emulsion stabilizer: functional properties and effects on pancreatic lipolysis. Food Funct..

[45] Raja Mohd Hafidz R.N., Yaakob C.M., Amin I., Noorfaizan A. (2011). Chemical and functional properties of bovine and porcine skin gelatin. Int. Food Res. J..

[46] Shakila R.J., Jeevithan E., Varatharajakumar A., Jeyasekaran G., Sukumar D. (2012). Functional characterization of gelatin extracted from bones of red snapper and grouper in comparison with mammalian gelatin. Lebensm. Wiss. Technol..

[47] Roy B.C., Das C., Hong H., Betti M., Bruce H.L. (2022). Effects of dehairing treatment on gelatin yield and quality from bovine hides. Waste Biomass Valorization.

[48] Sila A., Martinez-Alvarez O., Haddar A., Gómez-Guillén M.C., Nasri M., Montero M.P., Bougatef A. (2015). Recovery, viscoelastic and functional properties of Barbel skin gelatine: investigation of anti-DPP-IV and anti-prolyl endopeptidase activities of generated gelatine polypeptides. Food Chem..

[49] Nurul A.G., Sarbon N.M. (2015). Effects of pH on functional, rheological and structural properties of eel (*Monopterus sp.*) skin gelatin compared to bovine gelatin. Int. Food Res. J..

[50] He J., Zhang J., Xu Y., Ma Y., Guo X. (2022). The structural and functional differences between three species of fish scale gelatin and pigskin gelatin. Foods.

[51] Koli J.M., Basu S., Kannuchamy N., Gudipati V. (2013). Effect of pH and ionic strength on functional properties of fish gelatin in comparison to mammalian gelatin. Fish. Technol..

[52] Ninan G., Jose J., Abubacker Z. (2011). Preparation and characterization of gelatin extracted from the skins of rohu (*Labeo rohita*) and common carp (*Cyprinus carpio*). J. Food Process. Preserv..

